# Production of carbon-containing pyrite spherules induced by hyperthermophilic Thermococcales: a biosignature?

**DOI:** 10.3389/fmicb.2023.1145781

**Published:** 2023-05-25

**Authors:** Chloé Truong, Sylvain Bernard, Pierre Le Pape, Guillaume Morin, Camille Baya, Pauline Merrot, Aurore Gorlas, François Guyot

**Affiliations:** ^1^Institut de Minéralogie, de Physique des Matériaux et de Cosmochimie (IMPMC), MNHN, CNRS, IRD, Sorbonne Université, Paris, France; ^2^CEA, CNRS, Institute for Integrative Biology of the Cell, Université Paris-Saclay, Gif-sur-Yvette, France; ^3^Institut Universitaire de France (IUF), Paris, France

**Keywords:** archaea, biosignatures, hydrothermal vents, pyrite, greigite

## Abstract

Thermococcales, a major order of hyperthermophilic archaea inhabiting iron- and sulfur-rich anaerobic parts of hydrothermal deep-sea vents, are known to induce the formation of iron phosphates, greigite (Fe_3_S_4_) and abundant quantities of pyrite (FeS_2_), including pyrite spherules. In the present study, we report the characterization of the sulfide and phosphate minerals produced in the presence of Thermococcales using X-ray diffraction, synchrotron-based X ray absorption spectroscopy and scanning and transmission electron microscopies. Mixed valence Fe(II)-Fe(III) phosphates are interpreted as resulting from the activity of Thermococcales controlling phosphorus–iron–sulfur dynamics. The pyrite spherules (absent in abiotic control) consist of an assemblage of ultra-small nanocrystals of a few ten nanometers in size, showing coherently diffracting domain sizes of few nanometers. The production of these spherules occurs via a sulfur redox swing from S^0^ to S^–2^ and then to S^–1^, involving a comproportionation of (-II) and (0) oxidation states of sulfur, as supported by S-XANES data. Importantly, these pyrite spherules sequester biogenic organic compounds in small but detectable quantities, possibly making them good biosignatures to be searched for in extreme environments.

## 1. Introduction

The activity of microorganisms may promote mineral dissolution and/or precipitation in hydrothermal mineral environments (e.g., Holden and Adams, 2003; Templeton et al., 2009; Houghton and Seyfried, 2010). Hydrothermal systems, in particular sulfur-rich hydrothermal vents, allow exchange of heat and chemical species between seawater and ocean rocks (Edmond et al., 1979; Stein and Stein, 1994; Elderfield and Schultz, 1996; Wheat et al., 2000). The mixture of the hot – up to 400°C – reduced fluid discharging from the vents with the cold – about 2°C – oxygenated sea water, results in the formation of chimneys accommodating very steep temperature and geochemical gradients (Tivey, 1995; Von Damm, 1995; Charlou et al., 2002; Schmidt et al., 2007; Flores et al., 2011). Iron sulfide minerals, such as pyrite (FeS_2_) and chalcopyrite (CuFeS_2_), are predominant in the inner and hotter parts (>250°C) of active chimneys (Feely et al., 1994; Ludford et al., 1996). The cooler middle layers (80–150°C) of the chimneys are mainly composed of calcium and magnesium sulfate minerals, such as anhydrite (CaSO_4_), but contain iron sulfides such as pyrite and marcasite (FeS_2_) as well (e.g., Langmuir et al., 1997; Schrenk et al., 2003; Rouxel et al., 2004). It has been proposed that those middle layers harbor a population of hyperthermophilic archaea (Schrenk et al., 2003; Lin et al., 2016), probably mainly composed of sulfur-reducers Thermococcales (Takai et al., 2001; Prieur et al., 2004; Kormas et al., 2006).

Thermococcales could be an important contributor to the precipitation of minerals in the middle and external cooler layers dominated by anhydrite. Gorlas et al. (2018, 2022) reported that Thermococcales induce the formation of greigite (Fe_3_S_4_) nanocrystals and of great amounts of pyrite (FeS_2_) when they are cultivated in an iron and sulfur-rich synthetic medium simulating mineralizing hydrothermal fluids. These studies also showed that the production of pyrite only occurs in the cases where Thermococcales produce sulfur-rich vesicles (S(0)-vesicles), i.e., if they grow in a medium containing sulfur at zero valent state [S(0)] (Gorlas et al., 2015, 2018, 2022). In fact, fermentation-assisted by elemental sulfur reduction made by Thermococcales involves an NAD(P)H elemental sulfur oxidoreductase (NSR) enzyme (Liu et al., 2005; Kobori et al., 2010; Bridger et al., 2011; Herwald et al., 2013) and can lead to the rapid accumulation of elemental sulfur in the cytoplasm, as was reported for *Pyrococcus furiosus* exposed to high concentrations of elemental sulfur (>6.4 g/L) (Schut et al., 2007). The production of sulfur-rich vesicles could thus be seen as a detoxifying process, involving the sequestration of excess sulfur at oxidation state of (0) or close to (0) within the cell and its transport outside of the cell (Gorlas et al., 2015). This mechanism likely occurs in natural environments, since hydrothermal fluids are generally rich in polysulfides (Luther et al., 2001; Waite et al., 2008; Gartman et al., 2011) or colloidal reactive zero-valent sulfur.

In contact with an Fe (II)-rich fluid, these sulfur-rich vesicles could act as a precursor for pyrite formation, most likely after their release by the cells. In contrast, the production of greigite derives from the sulfurization of amorphous Fe (III) phosphates close to the surface of the cells (Gorlas et al., 2018, 2022). Although the excess of sulfide species (H_2_S and HS^–^) in the system should quickly convert greigite into pyrite (Posfai et al., 1998; Hunger and Benning, 2007), greigite was observed over a period ranging from a few days to several weeks in previous cultures (Gorlas et al., 2018, 2022). Because the stability of greigite depends on the balance between the abundance of reactive iron and the sulfide or polysulfide activities (Kao et al., 2004), its presence over rather long periods suggests that Thermococcales influence the reactivity of at least one, if not both, of these species. More data on the sequence of production and relative abundance of these mineral phases (pyrite, greigite, iron phosphates) in the presence of Thermococcales are needed to better understand the possible role of high temperature microorganisms in the mineralogy of hydrothermal systems. Special attention needs to be given to the habitus of these phases as identifying mineral phases with characteristics specific to the presence of Thermococcales (e.g., shape, size, crystallinity, content in organics), which could be used to track their presence in sulfur-rich hydrothermal vents.

To better understand how archaeal cells influenced the mineral environment and *vice versa*, this study focuses on the mineral characterization of the iron sulfides and iron phosphates produced in the presence/absence of *Thermococcus kodakarensis* in a medium containing zero-valent sulfur S(0). We determined the sequence of production and the habitus of the mineral phases produced in the cultures using X-ray diffraction (XRD), X-ray absorption (XAS) and electron microscopies (SEM and TEM). In addition to proposing a unified explanation of cell growth in strongly mineralized media, we documented the production of pyrite spherules. The specific shape and microstructure of these spherules possibly make them biosignature which presence in natural hydrothermal settings could be used to track the current or past activity of hyperthermophilic archaea.

## 2. Materials and methods

### 2.1. Mineralization process in anoxic conditions

*Thermococcus kodakarensis* KOD1 (JCM 12380) cultures were prepared under strictly anaerobic conditions under N_2_ atmosphere in an anoxic Jacomex™ glove box (<1 ppm O_2_), as described in Gorlas et al. (2018). Cultures were performed in glass serum vials set with rubber stoppers and aluminium caps. Cells were grown during 12 h at 85°C in 10 mL of a modified Ravot medium (containing, per liter of distilled water : 1 g NH_4_Cl, 0.2 g MgCl2.6H_2_O, 0.1 g CaCl_2_.2H_2_O, 0.1 g KCl, 0.83 g CH_3_COONa.2H_2_O, 20 g NaCl, 1 g yeast extract, 1 g tryptone, 3 g PIPES, 0.001 g resazurin and Na_2_S to reduce the medium at 0.05% (w/v) (final concentration) in presence of S(0) (1 g/L) in order to reach 10^8^ cells/mL^–1^. Then an anoxic solution of ferrous sulfate (FeSO_4_) was added to the cultures (leading to a final concentration of 5 mM) to induce iron mineral precipitation. The mineralized cultures were incubated for different durations determined in previous studies (Gorlas et al., 2018, 2022), namely 5 h for the presence of amorphous iron phosphates, 96 h corresponding to the formation of iron sulfides, 192 h for the demineralization process correlated to the presence of iron phosphates and 35 days to document a very long mineralization period.

Two control experiments were conducted at 85°C for 96 h (i.e., the duration required to observe the production of iron sulfides in the presence of cells): (1) a cell-free abiotic control consisting of the modified Ravot medium with S(0) 1 g/L, Na_2_S at 0.05% (w/v) (final concentration) and the FeSO_4_ solution at 5 mm (final concentration), and (2) a biotic control, consisting of *T. kodakarensis* cells grown in the modified Ravot medium with S(0) (1 g/L), Na_2_S at 0.05% (w/t) (final concentration), but without FeSO_4_ supplementation.

### 2.2. X-ray absorption near edge structure at the S K-edge

The sulfur speciation in the bulk samples was determined by X-ray absorption near edge structure (XANES) spectroscopy analysis at the S K-edge. Samples were prepared using centrifugation (15 mL of each sample were centrifugated at 5000 g for 10 min).

X-ray absorption near edge structure (XANES) was performed in fluorescence mode at the 4–3 beamline at the Stanford Synchrotron Radiation Light Source (SSRL, California, CA, USA) with a Hitachi™ HTA 4-element solid-state Si drift detector for the samples produced in our experiments or a PIPS detector for some of the concentrated reference samples. The incident energy was set up with a Si(111) monochromator and calibrated by measuring a thiosulfate reference (absorption edge at 2472 eV) between each sample holder during the experiment. Samples were shipped to SSRL within anoxic containers, and a few mg of pure solid powders were spread over sulfur-free tape, mounted into sample holders in a COY*™* glove box onsite, and analyzed at room temperature under He flow. Between 1 to 4 scans were collected for each sample. Data were calibrated and averaged using the SIXPACK software (Webb, 2005). Then, averaged spectra were normalized using the ATHENA software (Ravel and Newville, 2005).

For data analysis, a Linear Combination Fitting (LCF) procedure was conducted on the S K-edge XANES data with model compounds. LCF analysis of the XANES spectra at the S K-edge was performed using a custom-built program (Morin et al., 2003) based on the Levenberg-Marquardt minimization algorithm. Fit quality was estimated by a R-factor and a reduced chi-square, and the uncertainty on each fitting parameter was estimated to 99.7% confidence (3 sigma) [see Baya et al. (2021) for details]. The set of model compounds included biogenic nanocrystalline mackinawite (FeS), elemental sulfur [S(0)], synthetic nanocrystalline pyrite (FeS_2_), and synthetic nanocrystalline greigite (Fe_3_S_4_). For nano-mackinawite, elemental sulfur, and pyrite, the spectra of model compounds are given in Baya et al. (2021). Briefly, nano-mackinawite refers to a biogenic mackinawite synthesized by incubating *Desulfovibrio capillatus* with Fe(III)-citrate in Ikogou et al. (2017). A powder sample of α-sulfur S(0) was taken from the IMPMC chemical stocks, and pyrite was pure pyrite synthesized according to the protocol reported in Baya et al. (2021). The additional sample of nanocrystalline greigite (Fe_3_S_4_) was synthesized at ambient temperature in a glove box by mixing an appropriate volume of ferric chloride (FeCl_3_) and ferrous chloride (FeCl_2_) solutions with a sodium sulfide (Na_2_S) solution while gently stirring, and was then kept under magnetic stirring during 3 months until being dried under vacuum in the IMPMC glove box. This last sample contains traces of FeS.

### 2.3. Powder X-ray diffraction and Rietveld refinement

Sample preparation was carried out under N_2_ atmosphere in an anoxic Jacomex™ glove box (<1 ppm O_2_). Samples were prepared using centrifugation (5 mL of each sample were centrifugated at 5000 g for 10 min). The supernatant was discarded and the solid phase was vacuum-dried in an anoxic glove-box (no rinsing). Powder samples were placed on a zero-background Si wafer and inserted in a custom-built anoxic sample chamber equipped with a Kapton^®^ window. The sealed chamber was then removed from the glove-box and XRD patterns were collected using an XPert Pro Panalytical diffractometer. Data were collected using Co Kα radiation in continuous scan mode with an equivalent 0.03°2 θ step counting 2.5 h per sample over the 5–100°2 θ. Scans were then shortened to the 10–100°2 θ because of the bump signal from the Kapton^®^ window at 7.2°2 θ. Rietveld analysis was performed with the xnd_1.3 code (Berar and Baldinozzi, 1998) using pseudo-Voigt line-shape profiles. Starting crystallographic parameters including space group, unit-cell parameters, atomic positions and isotropic Debye-Waller factors were taken from Rettig and Trotter (1987) for α elemental sulfur S(0), from Lennie et al. (1995) for mackinawite FeS, from Stanjek and Schneider (2000) for greigite Fe_3_S_4_ and from Bayliss (1977) for pyrite FeS_2_. The structure of β-Fe_2_PO_4_O from Ijjaali et al. (1990) was used for the barbosalite-like compound within the Fe_4_(PO_4_)_2_O_2_ – Fe_4_(PO_4_)_3_(OH)_3_ solid solution. Unit-cell and line-shape parameters were varied for major phases only. Iron occupation was refined for the barbosalite-like compounds in order to properly account for relative intensities. Scale factors were refined for all phases and were used to calculate relative weight fraction of the mineral phases in the samples using the classical procedure by Bish and Post (1993), assuming a sum of weight fractions equal to one.

### 2.4. Scanning electron microscopy coupled with energy dispersive X-ray spectroscopy

Sample preparation was carried out under N_2_ atmosphere in an anoxic Jacomex™ glove box (<1 ppm O_2_). 1 mL of each sample was filtered through a 0.2 μm polycarbonate filter in order to conserve the solid part of the samples (no rinsing). Filters were then deposited on a carbon tape and carbon-coated. SEM-EDXS data were collected at IMPMC, with a GEMINI ZEISSTM Ultra55 Field Emission Gun Scanning Electron Microscope equipped with a Bruker silicon drift detector for EDXS. Both images and EDXS data were collected using an acceleration voltage of 10 kV at a working distance of 7.5 mm.

### 2.5. Sample preparation by focused ion beam

Focused ion beam (FIB) foils (20 μm × 5 μm × 100 nm) were extracted from pyrite spherules using a FEI Strata DB 235 (IEMN, Lille, France). Milling at low gallium ion currents allowed minimizing common artifacts including local gallium implantation, mixing of components, redeposition of the sputtered material on the sample surface and significant changes in the speciation of carbon-based polymers (Bernard et al., 2009; Schiffbauer and Xiao, 2009).

### 2.6. Scanning transmission X-ray microscopy

Scanning transmission X-ray microscopy (STXM) analyses were performed on FIB foils to document the carbon speciation of the organics present within the pyrite spherules using the HERMES STXM beamline at the synchrotron SOLEIL (Saint-Aubin, France - Belkhou et al., 2015; Swaraj et al., 2017). Energy calibration was done using the well-resolved 3 p Rydberg peak of gaseous CO2 at 294.96 eV for the C K-edge. XANES hypercube data (stacks) were collected with a spatial resolution of 100 nm at energy increments of 0.1 eV over the carbon (270–340 eV) absorption range with a dwell time of less than 1 ms per pixel to prevent irradiation damage (Wang et al., 2009). Stack alignments and extraction of XANES spectra were done using the Hyperspy python-based package (De la Peña et al., 2018). Normalization of data was done using the QUANTORXS freeware (Le Guillou et al., 2018).

### 2.7. Transmission electron microscopy (TEM)

Samples were examined using a JEOL JEM-2100F at IMPMC, equipped with a field emission gun (FEG) operating at 200 kV. Mineral characterization was completed by selected-area electron diffraction (SAED) and high-resolution transmission electron microscopy (HRTEM).

## 3. Results

### 3.1. Optical appearance

**FIGURE 1:**
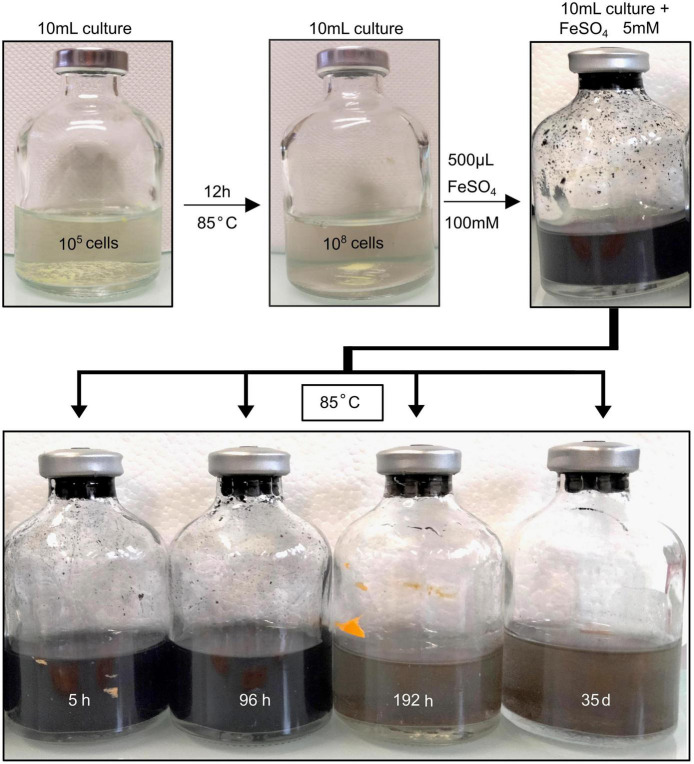
Schematic representation of the experimental protocol. *T. kodakarensis* cells were cultivated during 12 h in Ravot medium at 85°C which corresponds to the early stationary phase. Then, upon addition of aqueous FeSO_4_ solution (5 mM), mineralization occurred as observed visually. Mineralization experiments were conducted for 5 h, 96 h, 192 h, and 35 days. The mineral phases composing the solid residues were then studied by using a combination of XRD, SEM, XANES, TEM, and STXM. Two control experiments were conducted at 85°C for 96 h (i.e., the duration required to observe the production of iron sulfides in the presence of cells): (1) a cell-free abiotic control consisting of the modified Ravot medium with S(0) (1 g/L), Na_2_S at 0.05% (w/v) (final concentration), and the FeSO_4_ solution at 5 mM (final concentration) and (2) a biotic control, consisting of *T. kodakarensis* cells grown in the modified Ravot medium with S(0) (1 g/L), Na_2_S at 0.05% (w/v) (final concentration), but without FeSO_4_ supplementation.

Upon addition of ferrous sulfate (FeSO_4_) in the medium, abundant black precipitates were immediately generated both in abiotic controls (S(0)+Na_2_S+FeSO_4_) and in experiments conducted in the presence of cells (Figure 1). In presence of *T. kodakarensis*, the deep dark aspect of the precipitates faded after 192 h of mineralization (Figure 1), consistently with the observations reported in Gorlas et al. (2022). The abiotic controls retained their initial appearance over the entire duration of the experiments, no fading of the deep dark aspect of the precipitates occurred (Supplementary Figure 1).

### 3.2. Sulfur speciation

**FIGURE 2:**
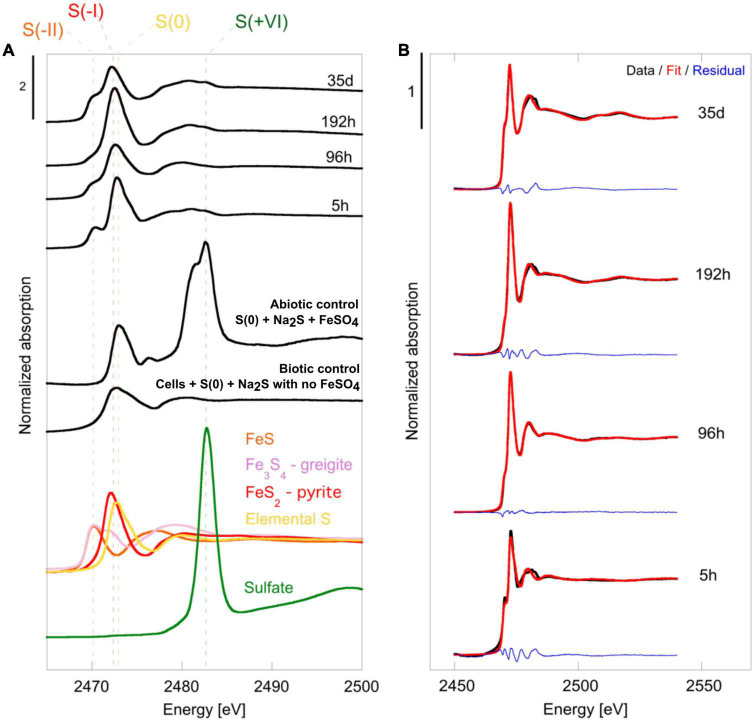
**(A)** Normalized S K-edge XANES spectra of selected reference compounds [FeS (S^–*II*^) in orange, Fe_3_S_4_ greigite in pink, FeS_2_ pyrite (S^–*I*^) in red, elemental sulfur S(0) in yellow and sulfate in green (S^+*VI*^)] of the cell-free abiotic control [S(0)+Na_2_S+FeSO_4_], of the biotic control [cells+S(0)+Na_2_S with no FeSO_4_] and of the solid residues of mineralization experiments conducted with *T. kodakarensis* in a sulfur and Fe^2+^ rich medium at 85°C for 5 h, 96 h, 192 h and 35 days. **(B)** Plot presenting linear combination fits performed on normalized S K-edge spectra of the 5 h, 96 h, 192 h and 35 days mineralization experiments conducted with *T. kodakarensis* (data in black, fit in red and residual in blue). Parameters relative to the LCF analysis, such as relative proportions of standard reference compounds and indicators of fit quality, are listed in Table 2.

X-ray absorption near edge structure (XANES) at the S K-edge of mineralized cultures of *T. kodakarensis* indicate a peculiar dynamic of sulfur redox evolution (Figure 2 and Table 1). The solid residues of the 5 h long mineralization experiments are dominated by the elemental sulfur [S(0)] introduced in the medium (63% (± 4) of the S atoms), nano-mackinawite (FeS) being also detected (38% (±4) of the S atoms). The proportion of sulfur as elemental sulfur corresponds to 60% (±3) of the S atoms in the solid residues of the 96 h long mineralization experiments. Nano-mackinawite is not present, while greigite (Fe_3_S_4_) and pyrite (FeS_2_) represent 25% (±1) and 14 (±3) of the S atoms, respectively. In the solid residues of the 192 h long mineralization experiments, the proportion of elemental sulfur only corresponds to 43% (±8) of the S atoms, while the proportion of sulfur as greigite corresponds to 19% (±3) of the S atoms and that of sulfur as pyrite to 40% (±8) of the S atoms. The solid residues of the 35 days long mineralization experiments do not contain any elemental sulfur, and sulfur is distributed between greigite (53% (±2) of the S atoms) and pyrite (46% (±2) of the S atoms). The solid residues of the abiotic control (S(0)+Na_2_S+FeSO_4_) and of the biotic control (cells+S(0)+Na_2_S with no FeSO_4_) (Figure 2) do not contain nano-mackinawite, greigite nor pyrite according to XANES data at the S K-edge. Although they are detected in the solid residues of the abiotic control (Figure 2), sulfates are not detected in the solid residues of mineralized cultures nor in the biotic control due to a common ion effect or a lack of iron in these experiments. The non-indexed peaks at 2472 and 2481 eV (close to the sulfate peak) in the abiotic control could be attributed to thiosulfate (Fleet et al., 2005).

**TABLE 1:** Results of the LCF analysis applied to normalized S K-edge XANES spectra using chosen reference compounds (see the section “2. Materials and methods”).

Sample	FeS %	S(0) %	Fe_3_S_4_ %	FeS_2_ %	Sum	χ^2^_R_ (0.10^–4^)	R-factor (0.10^–5^)
5 h	38 (4)	63 (4)	–	–	101	40.9	80.8
96 h	–	60 (3)	25 (1)	14 (3)	99	3.8	7.7
192 h	–	42 (8)	19 (3)	40 (8)	101	19.1	36.5
35 days	–	–	53 (2)	46 (2)	99	16.3	33.3

Uncertainties on the reported values are given considering a 99% confidence interval. Fit quality is estimated by a reduced chi-square and a R-factor (see the section “2. Materials and methods”).

### 3.3. X-ray diffraction identification of the crystalline phases formed in the presence of Thermococcales

**FIGURE 3:**
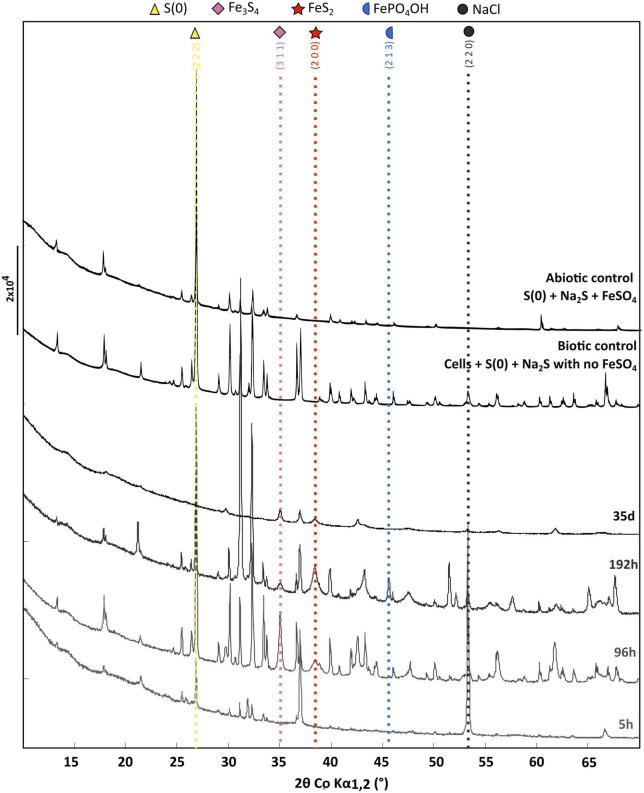
X-ray diffraction of the cell-free abiotic control [S(0)+Na_2_S+FeSO_4_], of the biotic control [cells+S(0)+Na_2_S with no FeSO_4_] and of the solid residues of mineralization experiments conducted with *T. kodakarensis* in a sulfur and Fe^2+^ rich medium at 85°C for 5 h, 96 h, 192 h and 35 days. For each phase, peaks corresponding to a line of significant intensity are labeled with concerned (h k l) of elemental sulfur (COD ID: 00-008-0247; yellow triangle), NaCl (halite COD ID: 00-005-0628; gray round), Greigite (COD ID: 00-016-0713; pink diamond), Pyrite (00-006-0710; red star) and Barbosalite-like (iron phosphate oxide hydroxide COD ID: 01-070-5888; blue moon).

X-ray diffraction patterns of the solid residues collected during the time-course mineralization experiments are displayed in Figure 3. After 5 h of mineralization, α-sulfur [S(0)] and halite (NaCl) are the major crystalline phases, halite having been likely crystallized upon drying (no rinsing). An additional broadened mackinawite (FeS) pattern is also detected and was included in the Rietveld analysis for this sample. After 96 h of mineralization, α-sulfur is still the dominant crystalline phase but pyrite (FeS_2_) and greigite (Fe_3_S_4_) are also observed in significant amounts, whereas halite is minor. After 192 h of mineralization, an iron (II)-(III) phosphate referred to as “barbosalite-like” is observed in large amount, in addition to pyrite and greigite. Based on Rietveld analysis it can be assigned to a member of the Fe^3+^_(4–x)_Fe^2+^_3x_(PO_4_)_3_(OH)_(3–3x)_O_3x_ solution (Schmid-Beurmann, 2000) with an *x* value of 0.28 as determined from iron occupancy-factor refinement, i.e., Fe^3+^_2.53_Fe^2+^_0.42_(PO_4_)_2_O_0.42_(OH)_1.58_ when compared to barbosalite (Fe^3+^)_2_Fe^2+^(PO_4_)_2_(OH)_2_ (Redhammer et al., 2000). After 35 days of mineralization, greigite, pyrite and some halite were the sole crystalline phases (Figure 3). Neither elemental sulfur nor crystalline iron (II)-(III) phosphate were detected. Note that large crystals may have not been sampled during preparation. Neither greigite, nor pyrite, nor barbosalite-like iron phosphate were detected in the solid residues of the abiotic control (S(0)+Na_2_S+FeSO_4_) and in the biotic control (cells+S(0)+Na_2_S with no FeSO_4_) (Figure 3 and Supplementary Figure 2).

Rietveld refinement (Table 2) allowed us to determine weight fractions of the crystalline phases composing the residues of the mineralization experiments and of the abiotic control (S(0)+Na_2_S+FeSO_4_). Mackinawite was detected in the form of “nano-mackinawite” after 5 h of mineralization with mean coherent domain size (MCD) <3 nm (Table 3) and unit-cell parameters close to those of mackinawite (Lennie et al., 1995). In this sample, elemental sulfur and nano-mackinawite accounted for 22(±6) wt% and 31(±9) wt% of the crystalline phases, respectively. After 96 h of mineralization, greigite and pyrite were present in similar proportions, 23(±1) wt% and 21(±4) wt%, respectively, and nano-mackinawite could not be quantified because of a too low amount. After 192 h of mineralization, greigite accounted for only 4(±1) wt% of the crystalline phases, whereas the barbosalite-like iron (II)-(III) phosphate was present at 50(±3) wt% and pyrite at 29(±3) wt%. After 35 days of mineralization, the proportion of greigite represented 34(±6) wt% of the crystalline fraction, while pyrite represented 52(±11) wt%. In the abiotic control (S(0)+Na_2_S+FeSO_4_), elemental sulfur accounted for 84(±16) wt% and the amount of nano-mackinawite was qualitatively estimated at ≤16 wt% (Table 2). After 96 h of mineralization, Rietveld refinement (Table 3) indicated isotropic mean coherent domain (MCD) size of 61(±11) nm greigite, and slightly anisotropic MCD for pyrite, with (111)-plane pseudo-platelets of 14(±4) nm width and 10(±4) nm thickness. MCD of both greigite and pyrite did not significantly evolve through time.

**TABLE 2:** Relative weight fraction of the mineral phases determined by Rietveld analysis applied to samples XRD patterns using pseudo-Voigt line-shape profiles (see the section “2. Materials and methods”).

Sample	FeS %	S(0) %	Fe_3_S_4_ %	FeS_2_ %	NaCl	FePO_4_(OH)	Sum
5 h	31 (9)	22 (6)	–	–	47 (11)	–	100
96 h	–	52 (1)	23 (1)	21 (4)	4 (1)	–	100
192 h	–	12 (1)	4 (1)	29 (3)	5 (5)	50 (3)	100
35 days	–	–	34 (6)	52 (11)	12 (3)	–	100
Abiotic control S(0)+Na_2_S+FeSO_4_	16	84 (16)	–	–	–	–	100

**TABLE 3:** Mean coherent domain size of the mineral phases based on Rietveld analysis applied to samples XRD patterns.

Sample	FeS	Fe_3_S_4_	FeS_2_		FePO_4_(OH)
			**L_0_^2^**	**L_2_^2^**	
5 h	<3	–	−	–	–
96 h	–	61 (10.7)	14.1 (3.5)	10.5 (3.5)	–
192 h	–	41.6 (0.1)	15.7 (1.9)	9.3 (1.6)	>1000
35 days	–	46.5 (11.4)	12.5 (3)	5.7 (1.8)	–

Mineral particle size was calculated by using the Scherrer equation.

### 3.4. Electron and X-ray microscopies on the minerals produced in the presence of Thermococcales

#### 3.4.1. Nano-mackinawite (FeS)

**FIGURE 4:**
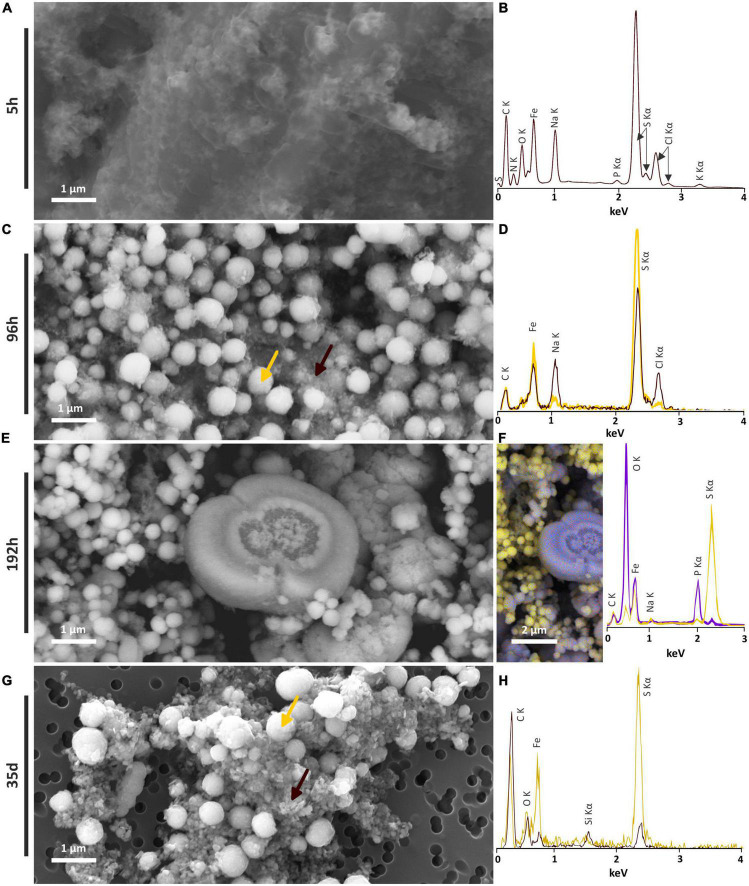
SEM investigations of the solid residues of mineralization experiments conducted with *T. kodakarensis* in a sulfur and Fe^2+^ rich medium at 85°C for 5 h, 96 h, 192 h and 35 days. **(A)** Iron sulfide matrix after 5 h of mineralization and **(B)** associated EDXS spectrum. **(C)** Iron sulfide spherules (indicated by a yellow arrow) and nanocrystals in the matrix (indicated by a brown arrow) after 96 h of mineralization and **(D)** associated EDXS spectra in yellow and brown, respectively. **(E)** Iron sulfide spherules and iron phosphate after 192 h of mineralization and **(F)** associated EDXS hypermap and spectra of iron phosphates (blue) and iron sulfides (yellow). **(G)** Iron sulfide spherules and crystallized iron sulfide matrix after 35 days of mineralization and **(H)** associated EDXS spectra in yellow and brown, respectively.

In the solid residues of the 5 h long mineralization experiments, an iron-sulfur-phosphorus amorphous or poorly crystalline material was observed, containing sometimes NaCl crystals, as well as carbon, nitrogen and potassium (Figures 4A, B). At longer times, this amorphous material disappears for the benefit of crystalline phases. Those observations are consistent with XANES and XRD results. A similar iron-sulfur-phosphorus amorphous material is also detected in the abiotic control [S(0)+ Na_2_S + FeSO_4_], but neither nitrogen nor potassium were detected (Supplementary Figure 3).

#### 3.4.2. Greigite nano-crystals (Fe_3_S_4_)

**FIGURE 5:**
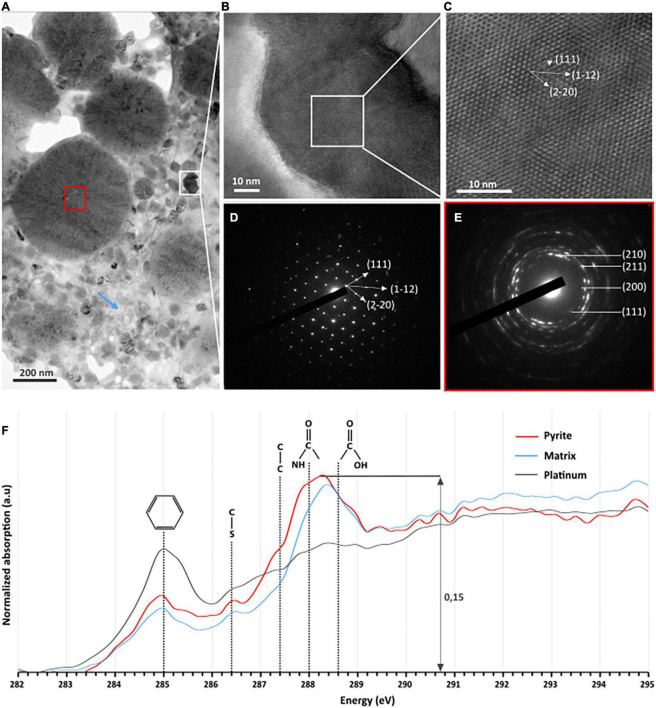
TEM, HRTEM, and STXM characterization of FIB sections of pyrite spherules observed in the solid residues of mineralization experiments conducted with *T. kodakarensis* in a sulfur and Fe^2+^ rich medium at 85°C for 96 h. **(A)** TEM image of sections of pyrite spherules. **(B)** TEM image and **(C)** HRTEM of greigite [zone axis ($1\,\bar{1}\,0$)] and **(D)** associated electron diffraction pattern. **(E)** Electron diffraction pattern of polycrystalline pyrite (red square in panel **A**) showing a preferential orientation. **(F)** C-XANES spectra of the organic material trapped into the pyrite spherules (red spectrum) and into the matrix surrounding the spherules (blue spectrum). The spectrum of the organic-rich platinum is also shown (in gray) for comparison. Absorption features at 285.0, 286.4, 287.4, 288.0, and 288.6 eV are attributed to aromatic groups, unsaturated C-S bonds, aliphatic groups, amide groups, and carboxylic groups, respectively.

Tiny greigite crystals are present in the solid residues of the 96 h long mineralization experiments (Figure 4C). These greigite crystals of about 40–60 nm are no longer present in the solid residues of the 192 h long mineralization experiments (Figure 4E), but crystals of greigite are present in the solid residues of the 35 days long mineralization experiments (Figure 4F). The nature of the nano-crystals as greigite was confirmed by Selected Area Electron Diffraction Pattern (SAED) and High Resolution TEM (HRTEM) images collected on the solid residues of the 96 h long mineralization experiments (Figures 5A–D).

#### 3.4.3. Pyrite (FeS_2_)

Submicrometric (from 200 nm to 1 μm) pyrite spherules are present in the solid residues of the 96 h, 192 h and 35 days long mineralization experiments (Figure 4). Their size, shape and smooth surface texture are very similar in all residues. Low magnification observations show homogeneous aggregates of pyrite spherules with relatively low disparities over the whole mineralization experiments (Supplementary Figure 3).

Focused ion beam (FIB) foils extracted from aggregates of pyrite spherules found in the solid residues of the 96 h long mineralization experiments reveal that each spherule is made of pure pyrite (Figure 5A). The SAED patterns reveal very tiny crystalline domains with some common orientations between adjacent domains (Figure 5E). These observations are consistent with Rietveld refinement of XRD data according to which pyrites are made of small anisotropic coherent domains of 15 nm by 10 nm (Table 3). Moreover, STXM characterization of pyrite sections reveal the presence of organic compounds trapped within the spherules and in the matrix surrounding the spherules (Figure 5F). Absorption features at 285.0, 286.4, 287.4, 288.0, and 288.6 eV can be attributed to aromatic groups, unsaturated C-S bonds, aliphatic groups, amide groups and carboxylic groups, respectively, (Le Guillou et al., 2018). The organic material that can be found in the matrix exhibits the same absorption features as the one trapped into the pyrite spherules (Figure 5F). The carbon amount is rather low (∼0.15 optical density units) but still detectable. As a comparison, the spectrum of the organic-rich platinum only shows a feature at 285.0 eV, attributed to aromatic groups (Le Guillou et al., 2018).

#### 3.4.4. Barbosalite-like (Fe^3+^_2.53_Fe^2+^_0.42_(PO_4_)_2_O_0.42_(OH)_1.58_)

Barbosalite-like crystals are only observed in the solid residues of the 192 h long mineralization experiments. They exhibit heterogeneous shapes and sizes (Figures 4E, F). Some are several micrometers wide and display a spherical or broken spherical shape, while some submicrometric ones display a spherule shape and are in direct contact with pyrite.

## 4. Discussion

### 4.1. Evolution of the system over time: phosphorus–iron–sulfur dynamics

**FIGURE 6:**
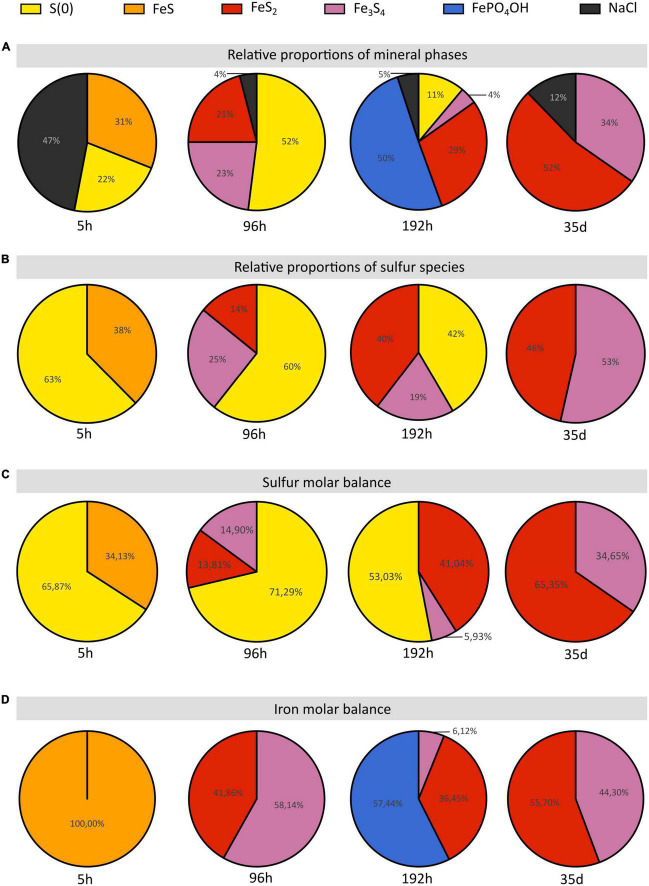
Proportions of sulfur and iron species and of the iron species of the solid residues of mineralization experiments conducted with *T. kodakarensis* in a sulfur and Fe^2+^ rich medium at 85°C for 5 h, 96 h, 192 h and 35 days. **(A)** Relative proportions of mineral phases according to Rietveld refinements. **(B)** Relative proportions of sulfur species according to S K-edge XANES data. **(C)** Sulfur and **(D)** iron molar balances based on Rietveld analyses (cf Table 2).

The formation of a black precipitate immediately after the addition in the medium of iron as Fe^2+^ (Figure 1) is caused by the precipitation of amorphous or poorly crystalline nanophases such as FeS nano-mackinawite [unambiguously detected by Rietveld refinement (Table 2) and XANES (Figure 2 and Table 1)] and iron phosphates (Figure 4B; Gorlas et al., 2018, 2022), the two phases forming a three-dimensional matrix observed by electron microscopy. A similar amorphous matrix is observed in the abiotic control [S(0) + Na_2_S + FeSO_4_] at 96 h (Supplementary Figure 3), identified as FeS nano-mackinawite by Rietveld refinement (Table 2). Note that the nano-mackinawite contribution, estimated at ≤16% ± while elemental sulfur accounted for 84 (± 16) wt %, likely is overestimated by Rietveld refinement (Table 2) since we were not able to detect it in XANES S K-edge analysis (Figure 2 and Table 1). Thermococcales promote a redox evolution of both sulfur and iron: S(0) is reduced by cellular metabolism producing sulfide (S^–2^) which is then progressively oxidized into S^–1^ as pyrite while Fe^2+^, although not directly involved in cellular metabolism, is partially oxidized into Fe^3+^ in greigite and in barbosalite-like phosphate (Figures 2, 6 and Tables 1, 2). In parallel with the continuous reduction of sulfur (0), the system thus evolves from almost pure nano-mackinawite (FeS) at 5 h (Figures 4A, B, 6) to greigite (Fe_3_S_4_) nanocrystals and pyrite (FeS_2_) submicrometric spherules starting 96 h (Figures 4C, D, 6).

While the precipitation of pyrite increases with increasing duration of mineralization (Figures 6A–C), the initial production of greigite, a sulfide containing 2 Fe(III) for 1 Fe(II), seems to be intimately related to that of Fe (II/III) phosphates in the present system: the proportion of greigite first decreases while barbosalite-like phosphates precipitate, before it increases once barbosalite-like are no longer present (Figures 6A, D). A number of studies have reported the microbial production of greigite either intracellularly (by magnetotactic bacteria for instance) or extracellularly (e.g., Mann et al., 1990; Bertel et al., 2012; Gorlas et al., 2018, 2022; Picard et al., 2018, 2019). Some authors proposed that the production of greigite requires a precursor already containing some Fe (III) (Etique et al., 2018; Picard et al., 2018; Berg et al., 2020; Duverger et al., 2020; Gorlas et al., 2022). This Fe(III) may come from Fe(III)-phosphates (e.g., Duverger et al., 2020) or from the oxidation of the Fe(II) of mackinawite (e.g., Lennie et al., 1997). Here, the FeS nano-mackinawite three-dimensional matrix contains some amorphous or poorly crystallized iron phosphates (Figure 4B), likely Fe(III)-phosphates as previously reported by Gorlas et al. (2022). Still, the initial production of Fe(III)-phases remains enigmatic since the experiments are conducted in strict anoxia. The oxidation of iron could have involved the S(0) contained in the cells, organic acids, or water (H^+^), which reductions could have been catalyzed by the cell surfaces. Kish et al. (2016) have reported that *Sulfolobus acidocaldarius* S-layer of both active and ghost cells and membrane vesicles are effective nucleation sites for amorphous or crystalline Fe-phosphate phases in a phosphate-rich and sulfate-rich medium. This possibility is also consistent with the observations of iron phosphates on Thermococcales cell surfaces or extracellular materials (Gorlas et al., 2022).

Between 96 and 192 h, the proportions of well crystallized pyrite and large iron phosphates, namely barbosalite-like [resembling barbosalite (Schmid-Beurmann, 2000) or lipscombite (Ech-Chahed et al., 1988)], increase over that of greigite (Figures 6A, D). The crystallized phosphates could be formed by interaction between poorly crystallized phosphates and greigite. Moreover, when sulfur is present in the medium, it has been shown that the cells accumulate S(0) vesicles leading to the formation of pyrite when in contact with Fe^2+^ (Gorlas et al., 2022), which explains the abundance of this phase. The predominance of such large grain size phases over nanophases of iron phosphate, nano-mackinawite and greigite likely explains the clarification of the medium (Figure 1 and Supplementary Figure 1). In similar 192 h long mineralization experiments, Gorlas et al. (2022) detected significant amounts of intracellular ATP and visualized living cells and cell divisions suggesting that some cells had resisted the toxic initial high nanoparticle-rich medium and benefited from clear enough medium to resume growth and cell division. It is then likely that these cells largely depleted the stock of sulfur (0) in the medium and shift to an H_2_ generating fermentative metabolism, below a certain threshold of zero valent sulfur in the system (Figure 6; Kanai et al., 2013; Schut et al., 2013). This may result in some pyrite dissolution producing H_2_S and greigite according to:


(1) $$3FeS_{2}+2H_{2}\to Fe_{3}S_{4}+2H_{2}S$$

and possibly iron (II)-(III) phosphates, which allow the cell population to recover additional phosphorus. Such phosphorus-iron-sulfur dynamics would constitute an ecological strategy in natural environments (Xiong et al., 2019; Wilfert et al., 2020). Such mobilization of the phosphorus reservoir by the cells leaves an excess of Fe(III) which can then be used for greigite precipitation. This model is consistent with the second phase of greigite precipitation (Figure 6) and the absence of well crystallized phosphates in the solid residues of 35 days long mineralization experiments (Figure 6).

### 4.2. Carbon-containing pyrite spherules: a biosignature?

In contrast to amorphous ferrous sulfide (FeS), greigite (Fe_3_S_4_) or mackinawite (FeS), which biological production has been extensively reported (Posfai et al., 1998; Picard et al., 2016, 2018; Stanley and Southam, 2018; Park and Faivre, 2022), pyrite is generally produced abiotically in natural settings (e.g., Yuan et al., 2020). Still, biogenic production of pyrite can be achieved by some microorganisms, including sulfate-reducing microorganisms (SRM) (Thiel et al., 2019; Berg et al., 2020; Duverger et al., 2020) or methanogenic archaea (Thiel et al., 2019). Pyrite may form from greigite and ferrous sulfide (Rickard, 1997; Hunger and Benning, 2007; Rickard and Luther, 2007) or from elemental sulfur and ferrous sulfide (Wilkin and Barnes, 1996; Benning et al., 2000). Here, an early ferrous sulfide phase is unambiguously detected by XANES (Figure 2 and Table 1), Rietveld refinement (Table 2) and SEM (Figures 4A, B), confirming previous results (Gorlas et al., 2018, 2022). This ferrous sulfide phase has likely been produced via interactions between S(-II) and Fe(II). The presence of both sulfides and hydrogen sulfide (HS^–^) results from the reduction of S(0) by *T. kodakarensis*, occurring partially before the addition of iron in the system (Morikawa et al., 1994). Note that it is likely that some sulfide ions come from the Na_2_S, explaining the production of black precipitates identified as ferrous sulfides by Rietveld refinement (Table 2) in the abiotic control (S(0) + Na_2_S + FeSO_4_) after addition of FeSO_4_.

As stated above, with increasing duration of mineralization, phases containing iron and/or sulfur more oxidized than mackinawite (FeS) are produced, namely greigite (Fe_3_S_4_), pyrite (FeS_2_) and barbosalite-like (Fe_1.47_PO_4_(OH)_0.79_) (Figures 2, 3, 4, 6). The sulfur of pyrite is at a formal oxidation state S(-I), i.e., it is more oxidized than that in mackinawite, which is formally S(-II), while both phases contain Fe(II). An oxidation process is thus necessary to form pyrite from mackinawite, i.e., electron acceptors must be present in the system. It is known that Thermococcales produce many extracellular vesicles (Soler et al., 2008; Gorlas et al., 2015; Liu et al., 2021), and particularly S(0)-vesicles which have been suggested to be involved in the detoxification of polysulfides (Gorlas et al., 2015). Here, the production of S(0)-vesicles may have enhanced the production of pyrite. Accordingly, Gorlas et al. (2015, 2018, 2022) have shown that no pyrite forms in culture devoid of S(0)-vesicles. Thus, the main process of pyrite formation in this system likely involves S(0)-vesicles, S(0) acting in such a scheme as an acceptor of sulfide electrons, according to:


(2) $$FeS+S\to FeS_{2}$$

The present study suggests that the presence of metabolically active *T. kodakarensis* influences the sulfur reactivity by producing S(0)-vesicles, which leads to a redox comproportionation of S(0) from elemental sulfur and S(-II) from FeS, to S(-I) in pyrite. Of note, using zero valent sulfur as an oxidant does not exclude greigite as a pyrite intermediate (Hunger and Benning, 2007).

The pyrite produced in the presence of Thermococcales present a peculiar spherical shape. The spherules with a diameter of 200 nm to 1 μm exhibit a very smooth surface texture (Figures 4C, E, G) and consist in an accumulation of many ultra-small domains sharing common preferential orientations in the spherules (Figure 5E). The ultra-small domains are anisotropic and about 15 nm by 10 nm (Table 3), which explains the very smooth aspect of the spherules. Moreover, the presence of complex organic matter is detected within these pyrite spherules, although in low quantity. These compounds exhibit several functional groups, including aromatic groups, unsaturated C-S bonds, aliphatic groups, amide groups and carboxylic groups (Figure 5F), i.e., typical of the functional groups measured in mineralization studies involving prokaryotes (Benzerara et al., 2006; Miot et al., 2009; Li et al., 2013, 2014; Picard et al., 2021).

Pyrite mineralization by Thermococcales has been proposed to constitute a survival strategy at the population level (Gorlas et al., 2022). Still, the production of the pyrite spherules described here may be bio-induced rather than bio-controlled. Frankel and Bazylinski (2003), described biological induced mineralization (BIM) as the unintended and uncontrolled result of metabolic products reacting with ions or compounds present in the environment, making BIM products rather difficult to distinguish from abiotic minerals (Beveridge, 1989; Konhauser, 1998; Banfield and Zhang, 2001; Bäuerlein, 2003). In contrast, biologically controlled mineralization (BCM) minerals leads to the production of structurally well-ordered, narrow size distributed minerals exhibiting specific morphologies (Frankel and Bazylinski, 2003; Liu et al., 2012). Given the homogeneity in texture, shape and size of the pyrite spherules discussed here, it seems that they rather correspond to BCM than to BIM. But BCM minerals are usually formed within intracellular organic matrices or vesicles, and their nucleation and growth are genetically controlled by the organism itself (Bazylinski and Frankel, 2000a,b), which is not the case here since pyrite most likely precipitates after the release of the S(0)-vesicles outside the cells. However, it could be argued that the BCM concept is relevant to the S(0)-vesicles themselves. Further studies, especially of the transcriptome, are necessary to determine if Thermococcales genetics are able to control the characteristics of pyrite spherules.

## 5. Conclusion

When cultivated in a ferrous and sulfur-rich medium, Thermococcales influence the reactivity of both species through iron sulfur and iron phosphate precipitation. After an initial precipitation of iron sulfide and phosphate nanophases that are toxic to most cells, the medium clears by evolving to the formation of larger structures of hundreds of nanometres pyrite spherules and well-crystallized iron II-III phosphates that are compatible with proper cell development. Moreover, this study shows that pyrite precipitation results from a redox comproportionation of S(0) (from elemental sulfur) and S(-II) (from FeS) to S(-I) (in pyrite), induced by the presence of Thermococcales and their production of S(0)-vesicles. Pyrites thus formed present specific textural features such as a peculiar spherule shape, ultra-small and anisotropic domains and a content in organic compounds that make them good candidates as biosignatures. However, before using them as tracers of the activity of Thermococcales in natural hydrothermal settings such as hydrothermal chimneys, additional experiments should be conducted to determine whether or not similar spherules containing similar organic compounds can be produced abiotically, and whether or not these specificities (shape, crystallinity and content in organics) may withstand hydrothermal and diagenetic conditions over long durations.

## Data availability statement

The original contributions presented in this study are included in the article/Supplementary material, further inquiries can be directed to the corresponding author.

## Author contributions

CT, AG, SB, and FG contributed to the conception and design of the study. CT and AG conducted the Thermococcales cultures, the mineralization process in anoxic conditions, and the powder X-ray diffraction. CT and FG conducted the electron microscopy analyses. PL, GM, CB, and PM conducted the XANES S K-edge measurements. GM realized the Rietveld refinement. CT and SB conducted the STXM analyses. CT wrote the first draft of the manuscript. CT, SB, FG, GM, and PL wrote the sections of the manuscript. All authors contributed to the manuscript revision, read, and approved the submitted version.

## Supplementary material

Supplementary Figure 1 **(A)** Mineralization experiments conducted with *T. kodakarensis* in a sulfur and Fe**^2+^** rich medium at 85°C for 192 h. **(B)** Abiotic control [S(0) + Na_2_S + FeSO_4_] after 192 h of mineralization at 85°C.

Supplementary Figure 2 X-ray diffractograms of the solid residues of mineralization experiments conducted with *T. kodakarensis* in a sulfur and Fe^2+^ rich medium at 85°C for 5 h **(A)**, 96 h **(B)**, 192 h **(C)** and 35 days **(D)** and of abiotic control [S(0) + Na_2_S + FeSO_4_] **(E)** and of biotic control (cells + S(0) + Na_2_S with no FeSO_4_) **(F)**. Each identified peaks are labeled with Elemental sulfur (COD ID: 00-008-0247; yellow triangle), NaCl (halite COD ID: 00-005-0628; gray round), Greigite (COD ID: 00-016-0713; pink diamond), Pyrite (00-006-0710; red star) and Barbosalite-like (iron phosphate oxide hydroxide COD ID: 01-070-5888; blue moon).

Supplementary Figure 3 Low magnification SEM images of the solid residues of mineralization experiments conducted with *T. kodakarensis* in a sulfur and Fe^2+^ rich medium at 85°C for 5 h **(A)**, 96 h **(B)**, 192 h **(C)**, 35 days **(D),** and of abiotic control [S(0) + Na_2_S + FeSO_4_] after 96 h of mineralization **(E)**.
