# Supplementary figures and images for: Production of carbon-containing pyrite spherules induced by hyperthermophilic Thermococcales: a biosignature?
Source: Front Microbiol. 2023 May 25;14:1145781. doi: 10.3389/fmicb.2023.1145781 (PMC10248028; doi:10.3389/fmicb.2023.1145781)

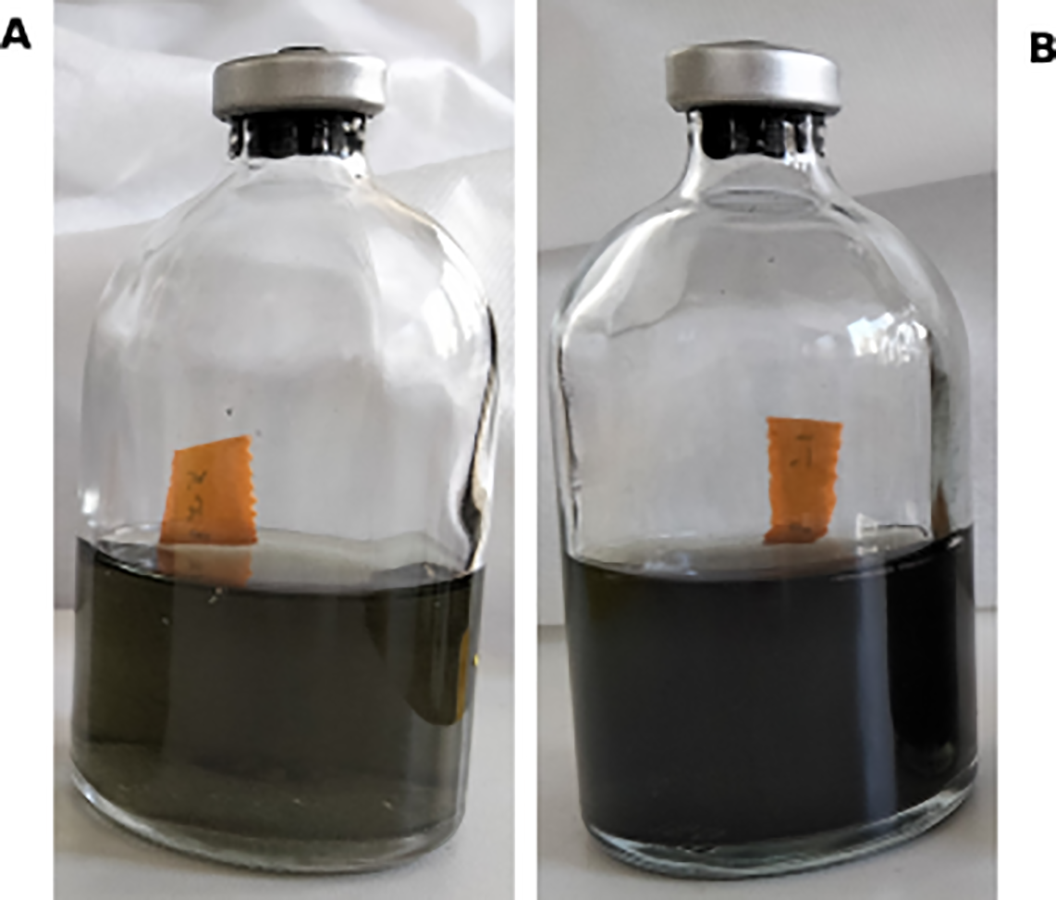

Supplement: Supplementary Figure 1 — (A) Mineralization experiments conducted with T. kodakarensis in a sulfur and Fe2+ rich medium at 85°C for 192 h. (B) Abiotic control [S(0) + Na2S + FeSO4] after 192 h of mineralization at 85°C. [file Image_1.TIFF]

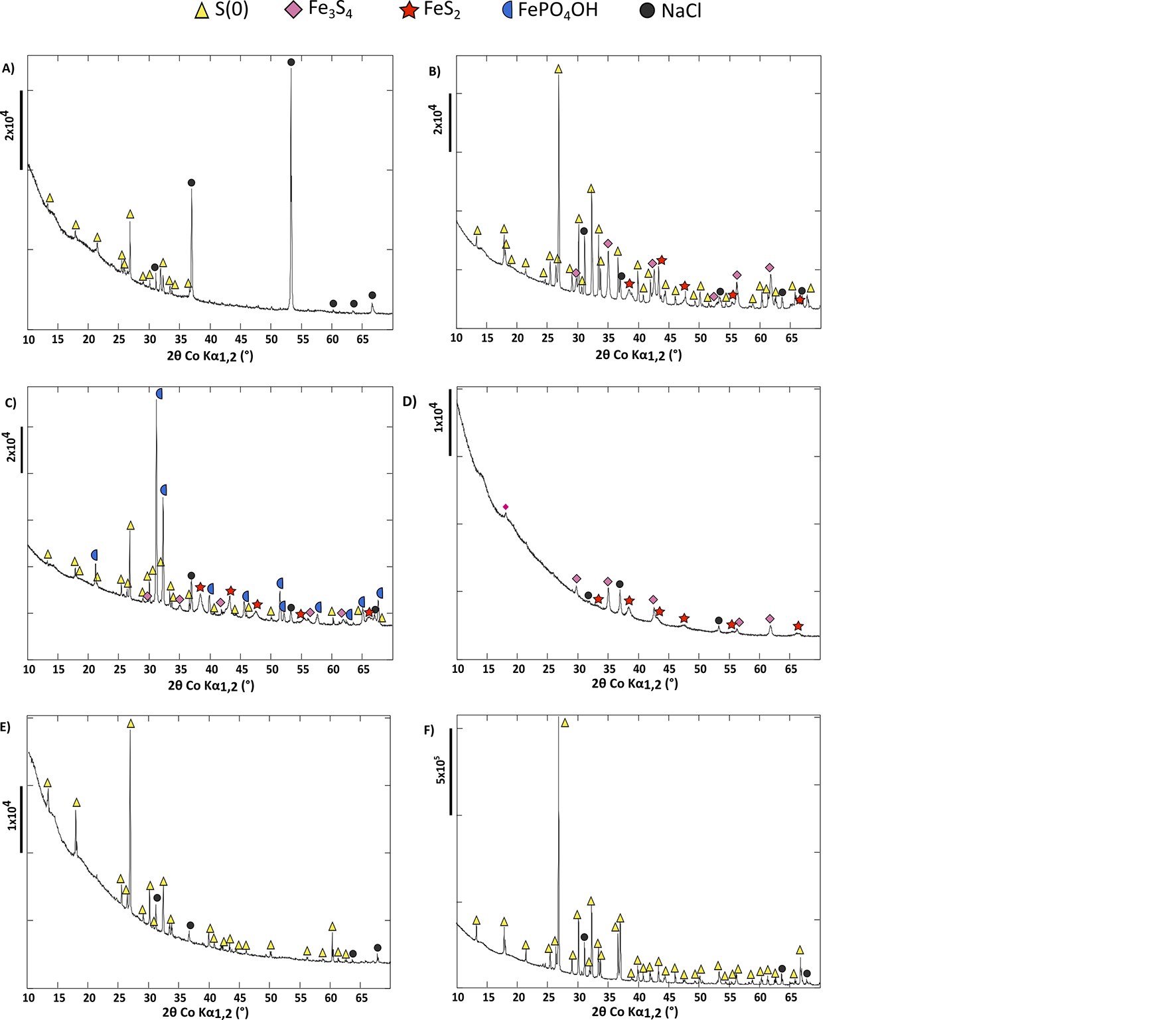

Supplement: Supplementary Figure 2 — X-ray diffractograms of the solid residues of mineralization experiments conducted with T. kodakarensis in a sulfur and Fe2+ rich medium at 85°C for 5 h (A), 96 h (B), 192 h (C) and 35 days (D) and of abiotic control [S(0) + Na2S + FeSO4] (E) and of biotic control (cells + S(0) + Na2S with no FeSO4) (F). Each identified peaks are labeled with Elemental sulfur (COD ID: 00-008-0247; yellow triangle), NaCl (halite COD ID: 00-005-0628; gray round), Greigite (COD ID: 00-016-0713; pink diamond), Pyrite (00-006-0710; red star) and Barbosalite-like (iron phosphate oxide hydroxide COD ID: 01-070-5888; blue moon). [file Image_2.TIFF]

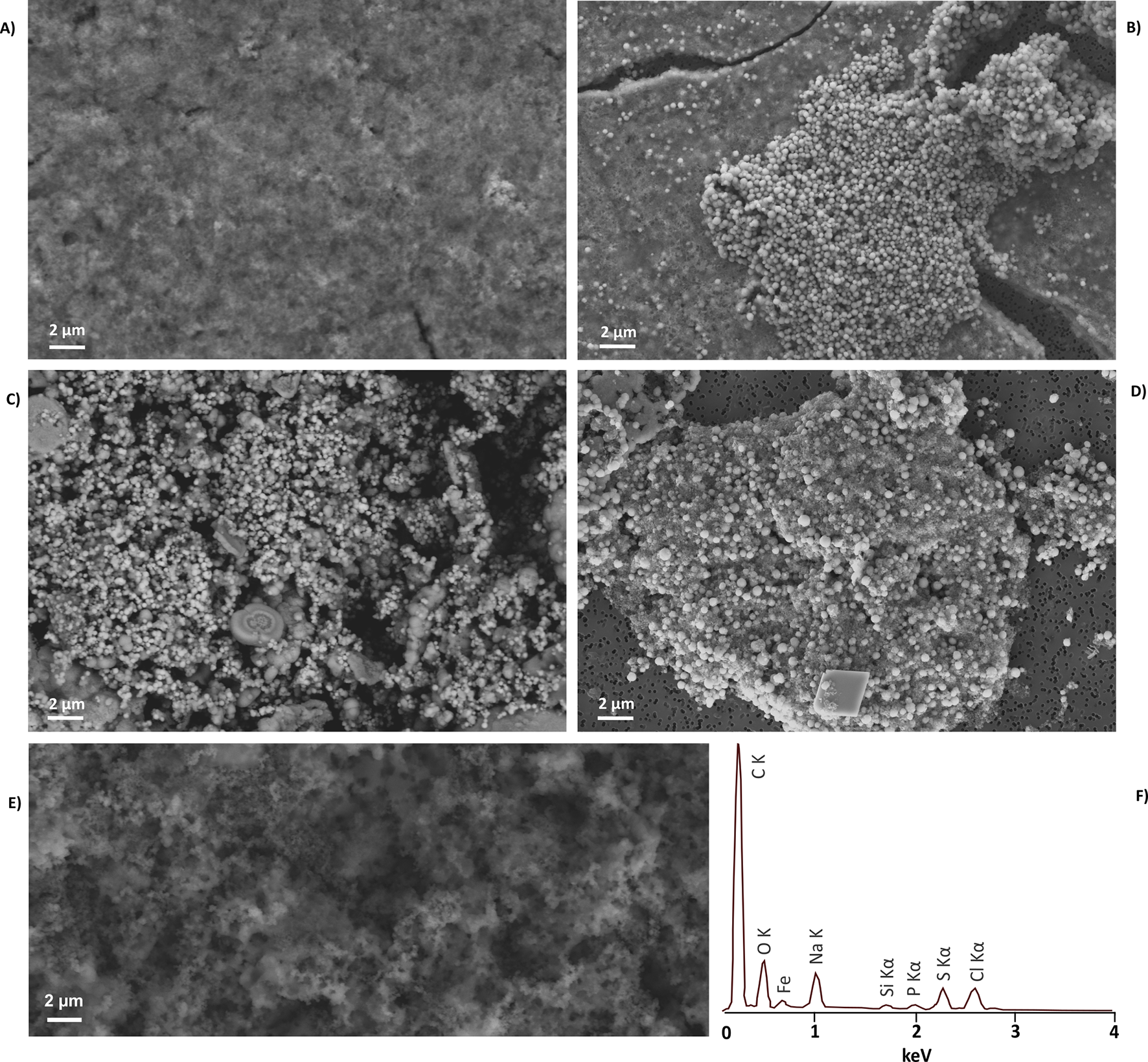

Supplement: Supplementary Figure 3 — Low magnification SEM images of the solid residues of mineralization experiments conducted with T. kodakarensis in a sulfur and Fe2+ rich medium at 85°C for 5 h (A), 96 h (B), 192 h (C), 35 days (D), and of abiotic control [S(0) + Na2S + FeSO4] after 96 h of mineralization (E). [file Image_3.TIFF]
